# ST-Elevation Myocardial Infarction in a 56-Year-Old Man With Acute Myeloid Leukemia: A Case Report

**DOI:** 10.7759/cureus.50230

**Published:** 2023-12-09

**Authors:** Hasan K Kazma, Malak Fakih, Batoul Danash, Malek Mohammed

**Affiliations:** 1 Division of Cardiology, Bahman Hospital, Beirut, LBN

**Keywords:** st elevation, antiplatelet, primary percutaneous coronary intervention (pci), coronary thrombo-aspiration procedure, drug-coated balloon, leukapheresis, hyperleukocytosis, leukostasis, aml, myocardial infarction

## Abstract

Acute myeloid leukemia (AML), the most common form of acute leukemia, is an aggressive lethal hematological malignancy that mainly occurs in older adults with a slightly higher predominance in males. It is prompted by the clonal expansion of immature myeloid blasts in the bone marrow, peripheral blood, and/or extramedullary tissues. Leukostasis in AML is a critical medical condition mainly affecting the lungs and brain and arises when tissue perfusion is compromised due to the clustering of white blood cells (WBCs) within the microvasculature. Cardiac involvement in this condition is exceptionally uncommon. Here, we present a case of a 56-year-old man, recently diagnosed with acute myelogenous leukemia M4 and leukostasis, who developed acute anterior ST-elevation myocardial infarction six days after presentation and in whom emergent coronary angiography showed proximal left anterior descending (LAD) artery lesion with a large clot obstructing the flow and thrombolysis in myocardial infarction (TIMI) I flow, and urgent percutaneous coronary intervention (PCI) was done; thromboaspiration and drug-coated balloon angioplasty were performed with good angiographic results. Antiplatelet (aspirin and clopidogrel) and anticoagulation (enoxaparin) were started immediately before PCI. Emergent leukapheresis was initiated in addition to hydroxyurea with complete resolution of chest pain. Four days post PCI, the patient developed right-sided hemiparesis with an evident infarct on a CT scan of the brain, and he also developed acute limb ischemia involving the distal right foot. Five days post PCI, the patient had a sudden sustained ventricular tachycardia followed immediately by asystole, and cardio-pulmonary resuscitation was done for 25 minutes but with no response.

## Introduction

Patients with acute myeloid leukemia (AML), hyperleukocytosis, defined as a WBC count greater than 50,000 or 100,000 cells/mL, and thrombocytopenia have a poor prognosis and high mortality rate [1]. Leukostasis, an acute medical emergency secondary to hyperleukocytosis, is typically due to blood hyperviscosity that affects mainly the lungs and the brain perfusion and to a lesser degree, other organs microvasculature, such as the heart [2,3]. The rare involvement of coronary arteries manifested by acute ST-elevation myocardial infarction accounts for 6% of cases with leukostasis and normal coronary angiogram, which can be treated with emergent leukapheresis, which can potentially lead to complete resolution of microvascular occlusion [4-6]. The concomitant AML and acute myocardial infarction are associated with very poor prognosis and high mortality rates [7]. In this case report, we present a rare case of AML complicated by an acute anterior ST-elevation myocardial infarction due to subtotal occlusion of an epicardial coronary artery, i.e., the LAD artery.

## Case presentation

**Figure 1:**
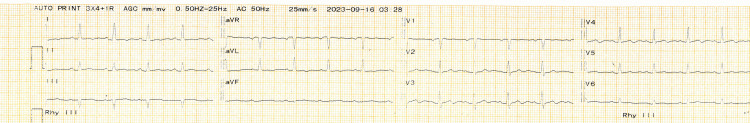
Normal baseline 12 leads ECG. ECG: electrocardiogram.

A 56-year-old man, a non-smoker, known to have hypertension on irbesartan 300 mg and hydrochlorothiazide 12.5 mg, presented with left thigh pain and swelling associated with chest discomfort, diaphoresis, palpitations, and dyspnea. He had an active lifestyle and did not report any recent long-distance travel, immobilization, or surgery. A review of his medical and clinical history showed notable weight loss but an absence of fever, night sweats, anorexia, or other constitutional symptoms. He mentioned an episode of diarrhea five days prior to the onset of symptoms. On examination, he was diaphoretic and in mild distress secondary to left leg pain and dyspnea. He had tachycardia with a heart rate of 108 beats per minute and he was febrile with a temperature of 38.8°C. Systolic blood pressure was maintained at 120 mmHg. Oxygen saturation by finger pulse oximeter was 96% on room air. Pulmonary and cardiac examinations were unremarkable. No jugular venous distension or hepato-jugular reflux was noted. However, he had left thigh pitting edema, erythema, hotness, and tenderness with adequate pedal and posterior tibial pulses bilaterally. Complete blood count with differential (CBCD) was notable for white blood cell (WBC) count of 61,500 cells/μL with 80% of abnormal cells and platelets count of 94,400 cells/μL. The electrocardiogram (ECG) on presentation was normal (Figure 1) with a negative troponin test.

**Figure 2:**
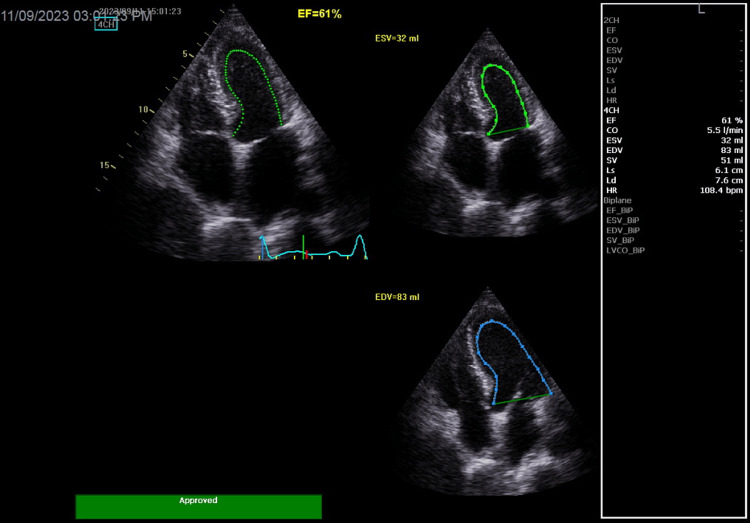
Baseline echocardiogram showing normal left ventricular ejection fraction at 61% with no wall motion abnormalities.

Echocardiography was normal and showed a left ventricular ejection fraction (LVEF) at 61% (Figure 2).

**Figure 3:**
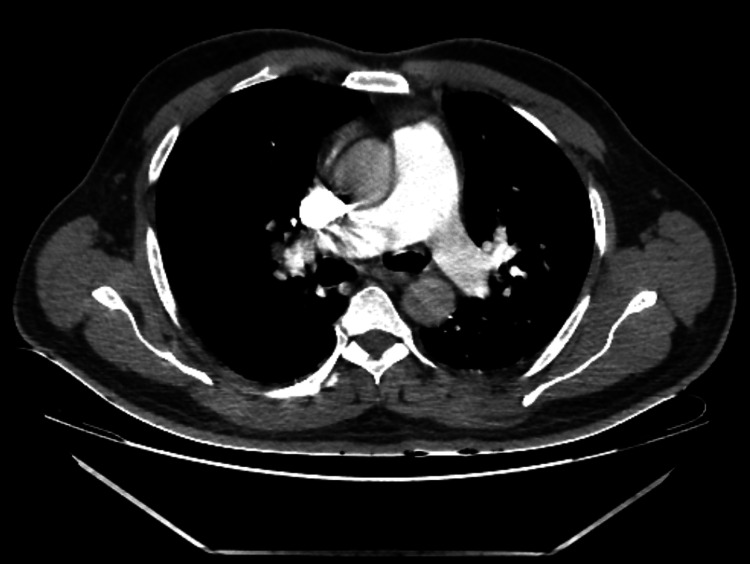
CT angiogram of the pulmonary arteries showing no evidence of pulmonary embolism.

CT angiography of the pulmonary arteries showed no evidence of pulmonary embolism (Figure 3) and a duplex scan of the left lower limb was negative for deep vein thrombosis but showed congested, edematous muscle of the anterior aspect of the left thigh suggestive of myositis.

**Figure 4:**
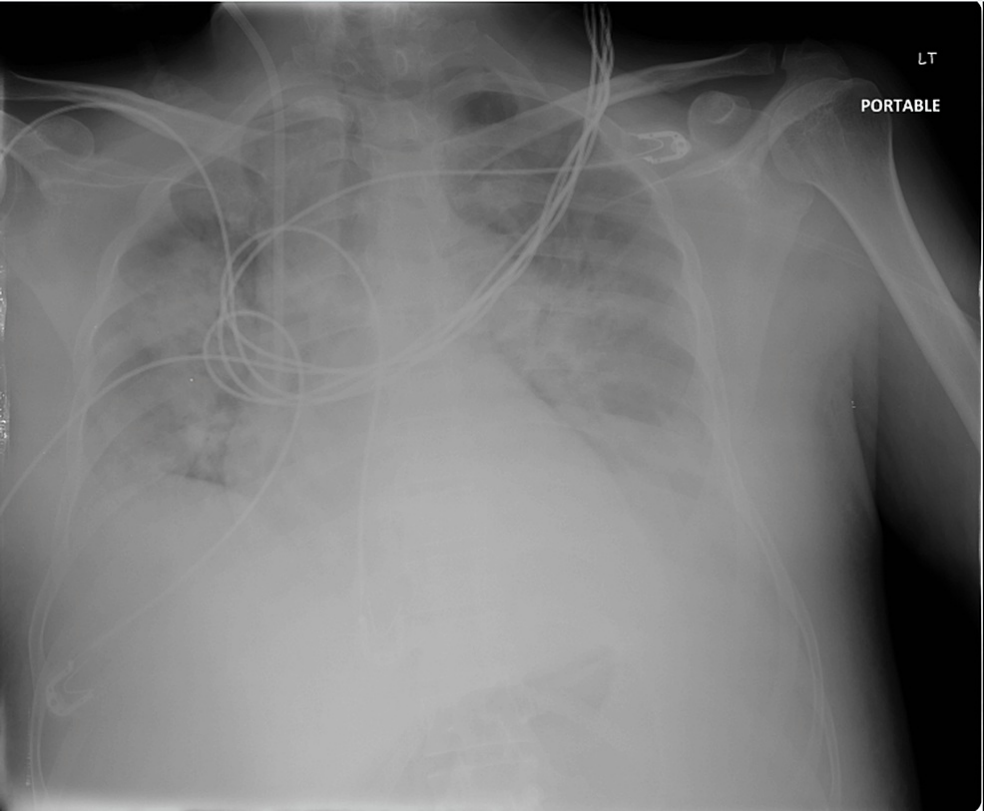
Chest X-ray showing bilateral lung infiltrates.

**Figure 5:**
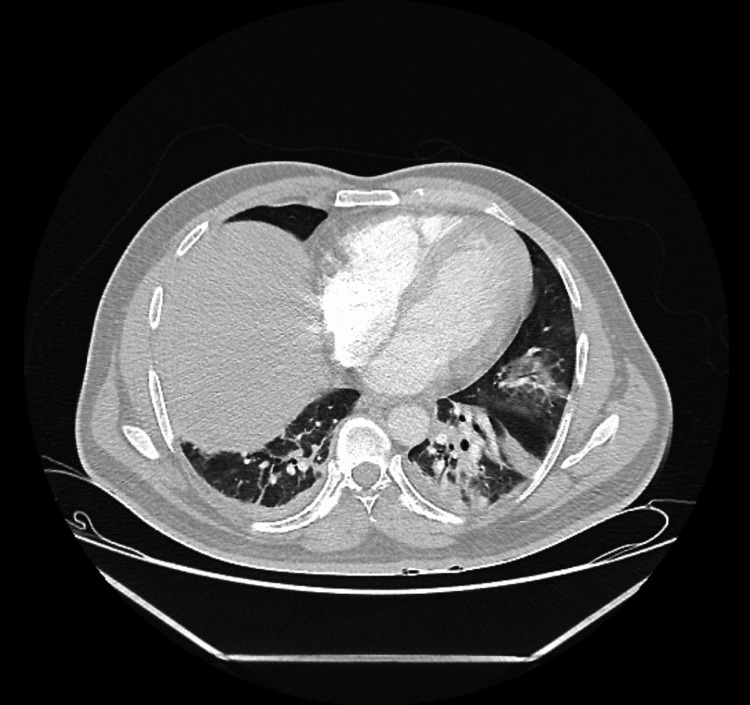
CT scan of the chest showing left lower lobe consolidation.

Leukemia was highly suspected and bone marrow aspirate with flow cytometry was positive for AML M4. The patient was started on hydroxyurea and allopurinol. Chemotherapy was postponed due to ongoing fever and pneumonia with bilateral lung infiltrates on chest X-ray (Figure 4) and left lower lobe consolidation on CT scan of the chest (Figure 5), so the patient was treated with broad-spectrum antibiotics and antifungal awaiting blood and urine culture results (vancomycin 1.2 g intravenously (IV) twice daily, meropenem 1 g IV three times daily, and voriconazole 300 mg IV twice daily). The patient also received deep vein thrombosis prophylaxis with subcutaneous enoxaparin 40 mg daily.

**Figure 6:**
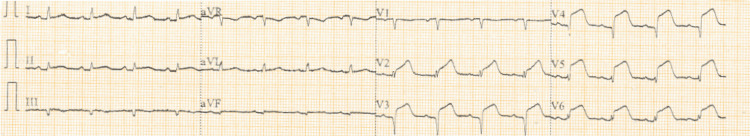
Twelve leads ECG done during chest pain immediately before coronary angiography showing ST elevation in anterior leads. ECG: electrocardiogram.

Subsequently, the WBCs continued to increase and by the sixth day of admission reached 201,000 cells/μL with a platelets count of 61,800 cells/μL. At that time, the patient had severe left-sided oppressive chest pain with marked ST segment elevation in anterior leads on ECG (Figure 6) and troponin level was 17.26 ng/ml (normal 0.035 ng/ml). Systolic blood pressure dropped to 90 mmHg.

**Figure 7:**
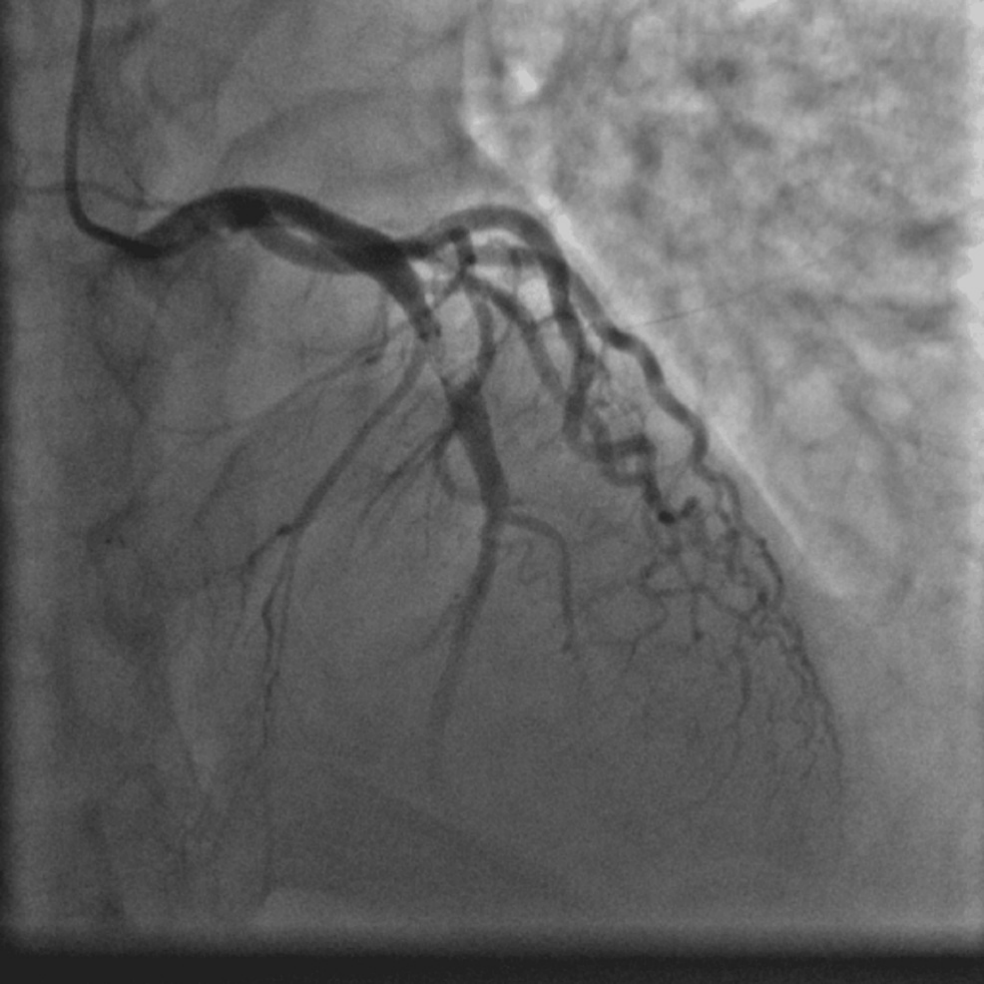
Coronary angiography showing 99% proximal LAD artery stenosis with a large clot and TIMI I flow. LAD: left anterior descending; TIMI: thrombolysis in myocardial infarction.

**Figure 8:**
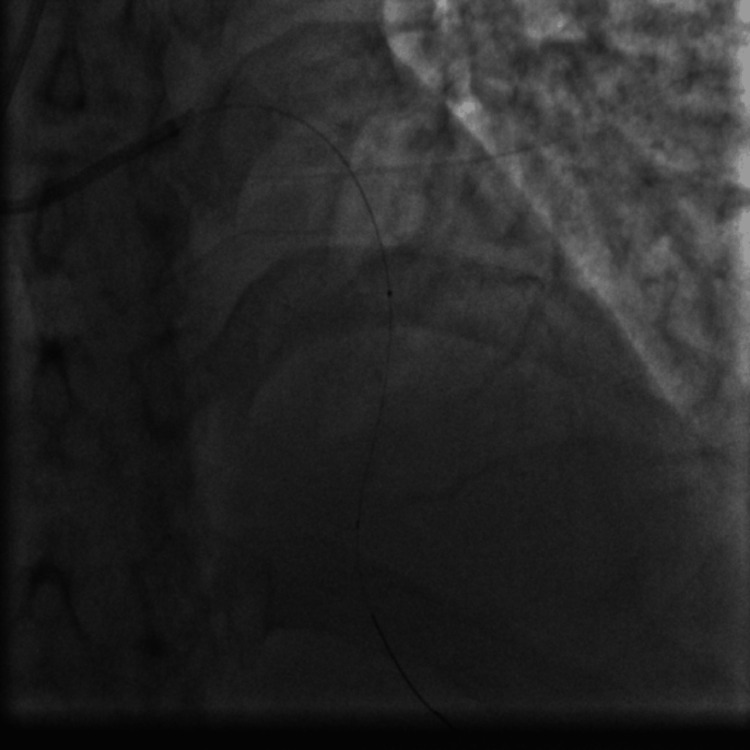
PCI using the Export Advance aspiration catheter (Medtronic, Minneapolis, MN) to manually aspirate the clot with a syringe. The dot seen on fluoroscopy represents the tip of the aspiration catheter inside the LAD at the thrombus site. PCI: percutaneous coronary intervention; LAD: left anterior descending.

**Figure 9:**
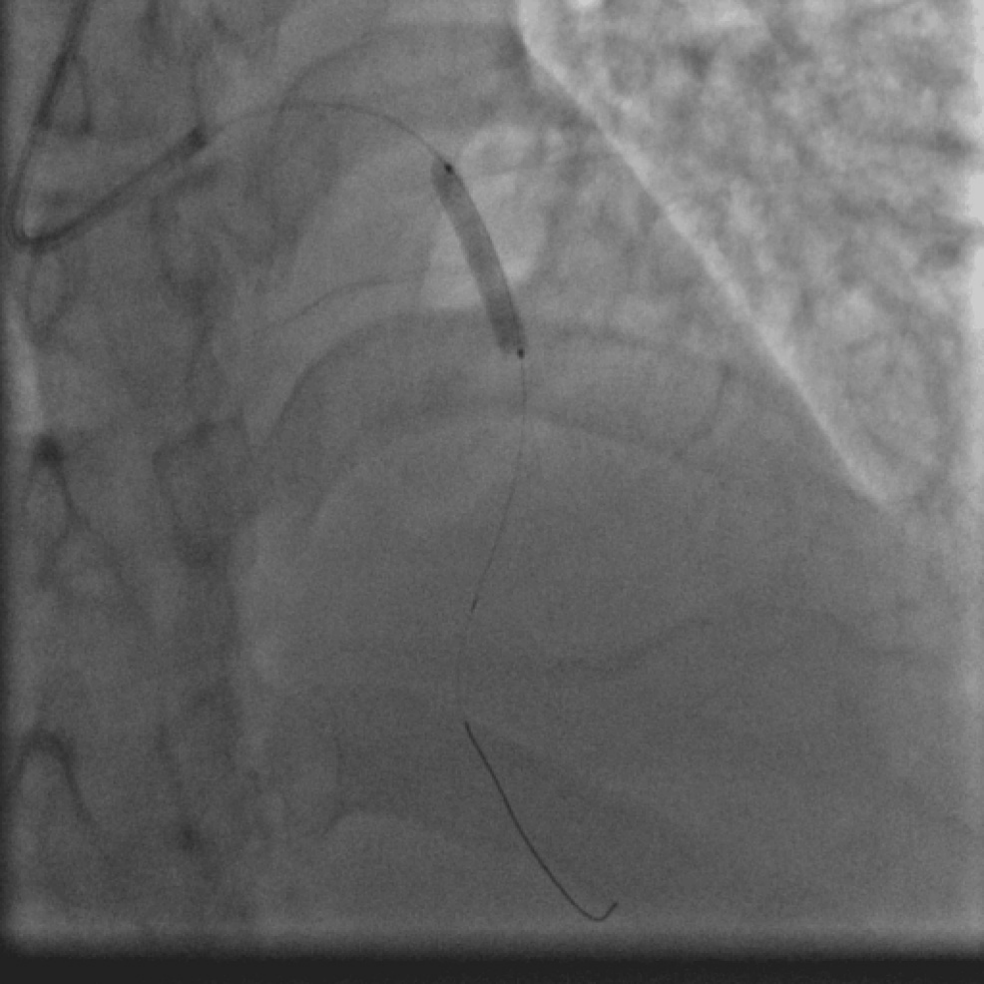
PCI using an Alex DCB to perform an angioplasty to the proximal LAD artery lesion after thromboaspiration. PCI: percutaneous coronary intervention; DCB: drug-coated balloon; LAD: left anterior descending.

**Figure 10:**
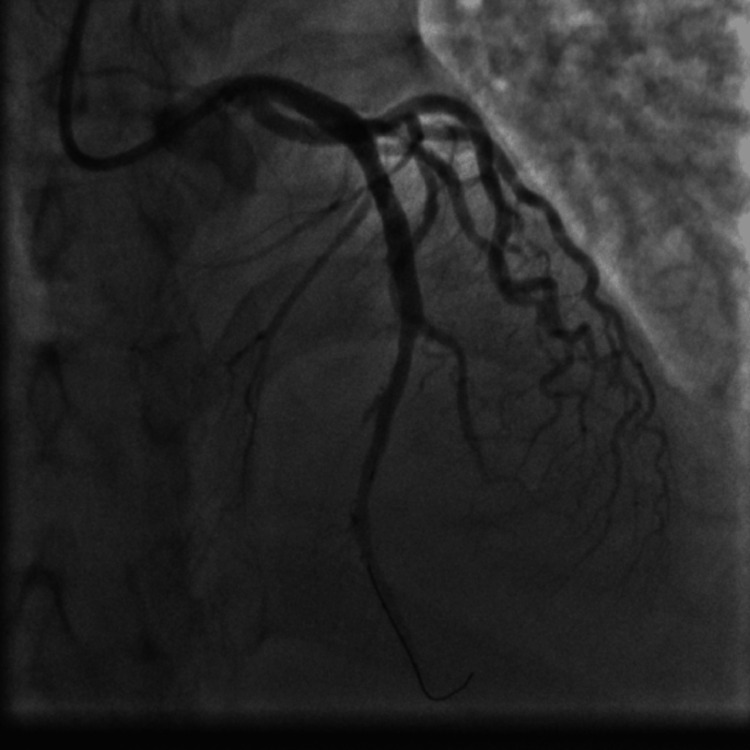
Coronary angiogram after thromboaspiration and DCB angioplasty showing good angiographic results of the treated LAD artery lesion with TIMI III flow. DCB: drug-coated balloon; LAD: left anterior descending; TIMI: thrombolysis in myocardial infarction.

Emergent coronary angiography via right radial access was done. The left anterior descending (LAD) artery showed a 99% proximal stenosis due to a large clot and thrombolysis in myocardial infarction (TIMI) I flow (Figure 7). We decided to do an emergency percutaneous coronary intervention (PCI) to the LAD artery, so the patient received aspirin 100 mg orally, clopidogrel 300 mg orally, and enoxaparin 40 mg intravenously, and thrombus aspiration was done twice using the Export Advance aspiration catheter (Medtronic, Minneapolis, MN) (Figure 8) and yielded a white thrombotic material after filtration of the blood aspirated from the thrombus aspiration catheter (kit for filtration is provided with the thrombus aspiration device). This was followed by balloon angioplasty using a 4.0 x 30 mm Alex Sirolimus drug-coated balloon (DCB) inflated at 8 atmospheric pressure (Figure 9) and resulted in TIMI III flow (Figure 10). Because of the concern from a possible leucocyte thrombus and ongoing thrombocytopenia in this patient with AML and since there was no acute recoil or thrombus formation by angiography at the end of the procedure (Figure 10), we elected not to deploy a stent to the dilated segment of the LAD. The patient was transferred to the coronary care unit (CCU) for continuous ECG monitoring after the PCI procedure.

Before PCI, the patient developed hypotension with no evidence of clinical choke. Inotropes (dobutamine 3 μg/kg/minute and norepinephrine 0.1 μg/kg/minute) were started during PCI then tapered and stopped over the next two days when the patient became hemodynamically stable. The patient was also suffering from fever and pneumonia, which could have contributed to sepsis and his hemodynamic status. After stopping inotropes, the patient remained stable hemodynamically. Aspirin 100 daily orally, clopidogrel 75 mg daily orally, and enoxaparin 40 mg daily subcutaneously were continued. The patient was also started on intravenous furosemide after PCI at a dose of 250 mg daily to treat pulmonary congestion; he required initially this high dose of furosemide to increase diuresis and this was due to the associated infection, sepsis, and hypotension. The dose of furosemide was subsequently tapered after a period of three days to 100 mg (IV) daily when the patient's hemodynamics had improved.

On the same day of PCI, leukapheresis was started and the leukocyte count decreased to 49,000 cells/µL after two sessions (two consecutive days of leukapheresis).

**Figure 11:**
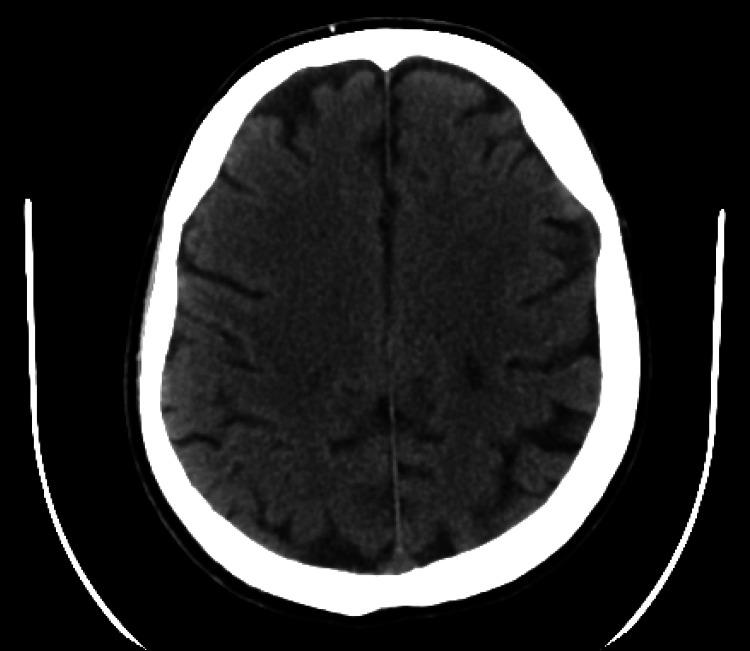
CT scan of the brain showing a 10 mm hypodense image in the posterior part of the semi-oval lobe consistent with a subacute ischemic process.

Chest pain resolved after PCI and the first session of leukapheresis; the left thigh pitting edema, erythema, hotness, and tenderness improved markedly after the second session of leukapheresis but the patient had persistent dyspnea and tachypnea with increased oxygen requirement due to pulmonary congestion, pneumonia, and possible lung leukostasis. Four days post PCI, the patient developed right-sided hemiparesis, and a CT scan of the brain was done immediately due to the concern from intracerebral hemorrhage (the patient was receiving dual antiplatelet therapy and anticoagulation prophylaxis and he had thrombocytopenia). A 10 mm hypodense image was seen in the posterior part of the semi-oval lobe on a CT scan of the brain (Figure 11), which was interpreted by our radiologist as a subacute ischemic process. Since the CT scan of the brain did not show the acute brain infarction until a few hours after the neurologic symptoms, the lesion seen on the CT scan of the brain was old. An MRI of the brain or a CT angiogram of the brain was not conducted because of the general condition of the patient and because we were not planning to perform a neuro-intervention.

**Figure 12:**
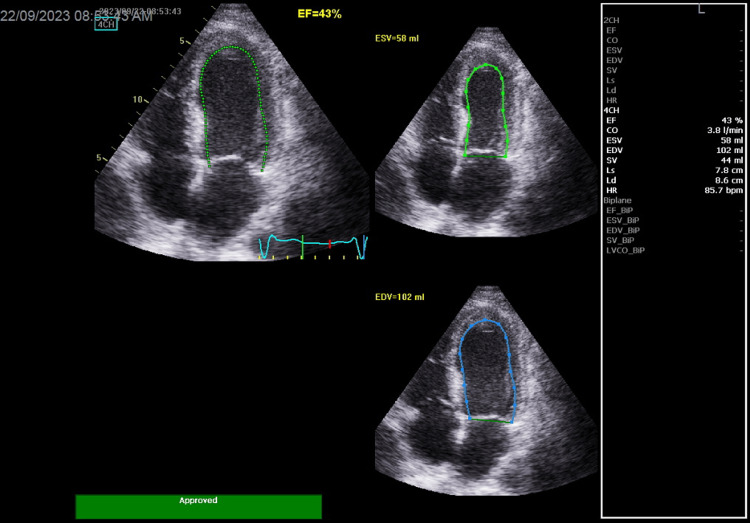
Echocardiogram done post MI and four days post PCI showing akinesia of the septum, apex, and mid-anterior segment with an LVEF at 43%. No left atrial and no left ventricular thrombi were noted. MI: myocardial infarction; PCI: percutaneous coronary intervention; LVEF: left ventricular ejection fraction.

In addition, he had an acute right distal limb ischemia with decreased right pedal and posterior tibial pulses by Doppler. Repeat echocardiogram done four days post PCI, after the cerebrovascular accident, revealed akinesia of the septum, apex, and mid-anterior segment, with overall ejection fraction at 43%; no left atrial (LA), no left ventricular (LV) thrombi, no masses, and no signs of impending cardiac rupture were detected on this study (Figure 12).

On the fifth day post PCI, the patient improved clinically and was hemodynamically stable without chest pain. He was in CCU under continuous ECG monitoring; 12 leads ECG was done on a daily basis and did not show any new findings. As mentioned above, the echocardiogram done four days post PCI (Figure 12) did not show any signs of impending cardiac rupture. The hematologist decided to start induction chemotherapy; however, before receiving chemotherapy, the patient developed sudden ventricular tachycardia followed immediately by asystole with no response after 25 minutes of resuscitation. Because the patient did not respond to resuscitation and the initial ventricular tachycardia degenerated rapidly into asystole, we considered cardiac rupture, massive pulmonary embolus, a new myocardial infarction, or the occlusion of the treated LAD lesion as possible causes of the cardiac arrest.

## Discussion

AML is an aggressive hematological malignancy that occurs at all ages but predominantly in patients older than 60 years [8]. It is characterized by clonal expansion of primitive hematopoietic stem cells resulting in abnormal differentiation of myeloid cells and a high number of immature malignant cells causing bone marrow failure, hyperleukocytosis, anemia, thrombocytopenia that presents with fatigue, leukostasis, infections due to nonfunctional neutrophils and bleeding disorders in addition to tumor lysis syndrome (TLS), and disseminated intravascular coagulation (DIC) [9-11].

Hyperleukocytosis, defined by WBC greater than 100,000 cells/µL, occurs in 18% of patients with AML and it is associated with poor prognosis and life-threatening complications such as leukostasis, DIC, and TLS [2]. Leukostasis is a medical emergency caused by obstruction of microvasculature and ischemia of target tissue. It is a clinical diagnosis manifested by respiratory distress, acute kidney injury, and central nervous system (CNS) disorders, with rare involvement of coronary arteries [2,4]. A retrospective study done by Stahl et al. that studied organs affected by leukostasis, in patients with newly diagnosed AML and leukocyte count > 50,000 cells/µL, discovered that the lungs and CNS are the most common organs involved with the incidence of 44% and 36%, respectively. On the other hand, cardiac involvement was reported in only 6% of cases, especially with normal coronary angiograms [4,12].

Symptomatic leukostasis is typically treated with leukapheresis until it is secure to start chemotherapy. Leukapheresis is the removal of white blood cells from the blood by the apheresis machine via centrifugation and reduction of the number of blast cells [2].

A study done by Villgran et al. regarding leukapheresis suggests that it could prevent further complications of hyperleukocytosis in newly diagnosed patients with AML until starting chemotherapy [13]. On the contrary, Choi et al. demonstrated no effect on survival outcomes and incidence of hyperleukocytosis-related complications such as DIC and TLS [14].

Our patient’s presentation of ST elevation myocardial infarction with a large clot on angiography causing obstruction of an epicardial coronary artery (LAD) was unusual due to rare involvement of epicardial coronary arteries in patients with AML and he was treated with thrombus aspiration and drug-coated balloon angioplasty with good angiographic result to the epicardial LAD. Thrombus aspiration did return a white-colored thrombotic material; this made us suspect that this lesion was due to AML during the procedure, so we elected not to deploy a stent (bare metal or drug-eluting stent) to the proximal LAD lesion because of the inherent risk of thrombocytopenia that develops in AML and the need to continue dual antiplatelet therapy in case of stent placement; also the LAD lesion remained stable with no recoil or clot formation in the vicinity of the treated LAD segment by the end of the procedure. We were also concerned, during the procedure, that AML as a disease can also predispose to stent thrombosis [7]. The patient was receiving dual antiplatelet therapy and anticoagulation prophylaxis, so acute thrombosis (platelet aggregation with activation of clotting cascade) of the treated LAD lesion leading to cardiac arrest is less likely but still a possibility [7]; however, a leucocytes clot at the treated LAD site is possible and may have led to the cardiac arrest. The echocardiogram done after the cerebral infarct and right lower limb ischemia did not show any thrombus in the LA or the LV apex, making an embolic process less likely but again an embolic process was still possible; finally, the multiple organ or artery involvement may have been due to leucostasis.

## Conclusions

AML is a fatal hematological malignancy manifested by life-threatening complications, such as leukostasis, resulting in obstruction of microvasculature that compromises tissue perfusion resulting in ischemia and end-organ damage. Myocardial infarction is an uncommon manifestation that presents typically with acute chest pain in patients with hyperleukocytosis. In the case of only microvasculature involvement, leukapheresis remains the gold standard treatment for symptomatic patients. It can potentially lead to complete reperfusion of microvasculature until it is safe to start induction chemotherapy. Our case focuses attention on the importance of diagnosis and rapid intervention and treatment of uncommon manifestations of AML: when the epicardial coronary artery is occluded. Also, it focuses on using thrombus aspiration and DCB angioplasty in AML with ST-elevation myocardial infarction when angiography reveals a large thrombus: thrombus aspiration may be done first followed by DCB-only angioplasty. If the angiographic results remain stable with no recoil or thrombus in the vicinity of the lesion, the procedure can be finalized without stent deployment to minimize the antiplatelet therapy duration in AML because of the inherent risk of thrombocytopenia in this disease; we need to consider also the fact that AML may increase the risk of stent thrombosis. However, if after DCB angioplasty the lesion still shows obstruction to flow, then a stent may be deployed to improve results and outcome. Intravascular imaging like intravascular ultrasound or optical coherent tomography can definitely guide the PCI procedures of these patients. Due to the rapid progression and death of our patient and unresponsiveness to resuscitation, cardiac free wall rupture, pulmonary artery occlusion, a new myocardial infarction, or re-occlusion of the treated LAD segment due to leukostasis or thrombus are possible causes of the sudden cardiac arrest.
